# Divergent branches of mitochondrial signaling regulate specific genes and the viability of specialized cell types of differentiated yeast colonies

**DOI:** 10.18632/oncotarget.8084

**Published:** 2016-03-15

**Authors:** Kristýna Podholová, Vítězslav Plocek, Stanislava Rešetárová, Helena Kučerová, Otakar Hlaváček, Libuše Váchová, Zdena Palková

**Affiliations:** ^1^ Department of Genetics and Microbiology, Faculty of Science, Charles University in Prague, Prague, Czech Republic; ^2^ Institute of Microbiology of the CAS, v.v.i., Prague, Czech Republic

**Keywords:** mitochondrial retrograde signaling, development and differentiation, ageing and longevity

## Abstract

Mitochondrial retrograde signaling mediates communication from altered mitochondria to the nucleus and is involved in many normal and pathophysiological changes, including cell metabolic reprogramming linked to cancer development and progression in mammals. The major mitochondrial retrograde pathway described in yeast includes three activators, Rtg1p, Rtg2p and Rtg3p, and repressors, Mks1p and Bmh1p/Bmh2p. Using differentiated yeast colonies, we show that Mks1p-Rtg pathway regulation is complex and includes three branches that divergently regulate the properties and fate of three specifically localized cell subpopulations via signals from differently altered mitochondria. The newly identified RTG pathway-regulated genes *ATO1*/*ATO2* are expressed in colonial upper (U) cells, the cells with active TORC1 that metabolically resemble tumor cells, while *CIT2* is a typical target induced in one subpopulation of starving lower (L) cells. The viability of the second L cell subpopulation is strictly dependent on RTG signaling. Additional co-activators of Rtg1p-Rtg3p specific to particular gene targets of each branch are required to regulate cell differentiation.

## INTRODUCTION

Mitochondrial retrograde signaling is a conserved mechanism that governs cell metabolic adaptation to altered mitochondrial functions, often preventing cell death, as described in mammals and yeast [1]. In both organisms, retrograde signaling can be triggered by changes causing a decrease in mitochondrial membrane potential (such as defects in respiratory chain components or mtDNA mutations) and by elevated reactive oxygen species (ROS) [2-4]. In mammals, various disorders often related to cancer have been linked with cell metabolic and physiological changes because of signaling from mitochondria. For example, retrograde signaling can cause pleiotropic cell changes linked with the upregulation of genes involved in tumorigenic pathways, such as the activation of different oncogenic factors, regulators and metabolic enzymes involved in the metabolic switch to aerobic glycolysis in tumor cells [4, 5]. A number of different factors in different mammalian cell types are involved in retrograde signaling; however, Ca^2+^ is assumed to be a key molecule at present [2, 4, 6].

The major mitochondrial retrograde signaling pathway described thus far in yeast includes three activators, Rtg1p, Rtg2p and Rtg3p, and is hence referred to as RTG pathway. Rtg1p and Rtg3p together form a heterodimeric transcription activator, the translocation of which from the cytosol to the nucleus is activated by Rtg2p [7]. Rtg2p mediates signals from mitochondria with reduced functions, e.g., due to various DNA mutations in the case of respiratory deficient mutants. Three negative regulators, Mks1p, Bmh1p and Bmh2p, which are involved in retaining the Rtg1p-Rtg3p complex in the cytosol, have been described [8, 9] as well as negative regulation of the RTG pathway *via* active TORC1 [10-12]. In addition, RTG gene-independent mitochondria-to-nucleus signaling has been proposed in yeast [13, 14]. Bioinformatics analysis has shown that the mammalian heterodimer Myc-Max, of which the basic helix-loop-helix leucine zipper transcription factor Myc is often activated during retrograde signaling, has structural homology to the Rtg1p-Rtg3p heterodimer [15]. Hence, the Myc-Max heterodimer, together with its upstream regulator NF-κB, is positioned in a retrograde signaling pathway in mammals in parallel to Rtg1p-Rtg3p in yeast [15, 16]. Homologs of Rtg2p have not yet been identified in metazoans. RTG signaling is linked with the metabolic reprogramming involved in yeast adaptation to mitochondrial dysfunctions by the activation of anaplerotic reactions and peroxisomal functions such as the glyoxylate cycle [13, 17]. The *CIT2* gene, encoding the peroxisomal isoform of citrate synthase, is the typical target gene whose expression is induced by RTG signaling [18].

Yeast colonies have become an excellent model for the investigation of processes involved in the differentiation of cells and the development of specific cell types [19]. When growing on solid complex respiratory medium, yeast giant colonies (colonies derived from a drop of cell suspension spotted on the agar) as well as microcolonies (colonies derived from single cells) pass through distinct developmental phases that can be detected by monitoring the pH changes of the medium, changing from the acidic to near alkali and vice versa [20-22]. The alkali phase of colony development is accompanied by the production of volatile ammonia that functions as a signal important for colony metabolic reprogramming and long term survival [20, 23, 24]. Two major cell types (U cells in upper regions and L cells in lower regions) have been identified in alkali-phase colonies [22, 25]. Both of these cell types originate from mostly non-dividing cell progenitors that form colonies in the acidic phase preceding the ammonia signaling period [25, 26]. U cells, which have a stress-resistant and longevity phenotype, active TORC1, active autophagy, ammonia production, aerobic glycolysis and high glutamine content, resemble mammalian tumor cells [25, 27]. In contrast, L cells exhibit features of starving cells; L cells are also sensitive to stresses and lose viability more quickly during colony aging than U cells. L cells potentially provide nutrients to U cells *via* a nutrient flow cycle similar to the Cori cycle and glutamine-ammonium cycle described between cells of solid tumors and other tissues of tumor-affected mammalian organisms [25, 27].

One of the prominent differences between U and L cells involves mitochondria and respiration. U cells, although localized to upper parts of colonies situated close to the air, decrease their capability to respire almost to the level typical of fermenting cells and harbor large swollen mitochondria [22, 25]. Reduced respiration could contribute to another typical feature of U cells, which is a negligible level of ROS in these cells. The ROS level in U cells is even lower than that in the cells of younger acidic phase colonies [24]. In contrast, L cells are capable of respiration and contain normal-looking mitochondria [22, 25]. The ROS level in L cells is elevated during the alkali period of colony development.

Here, we show that mitochondrial signaling is mediated by the three different branches of the RTG pathway that are involved in cell differentiation within the colonies, in the expression of specific genes and in the viability of particular cell subpopulations. We show that in addition to major U/L cell differentiation, smaller cell subpopulations are formed within L cells and that their survival depends differently on RTG pathway activity. In addition to *CIT2*, we identified two of the three *ATO* genes (*ATO1* and *ATO2*) involved in ammonia signaling as target genes regulated by one of the RTG signaling branches, and we identified the differences in the manner by which the RTG pathway regulates different genes in specific cell subpopulations within differentiated colonies. We also show that Mks1p, a negative regulator of the RTG pathway, contributes differently to divergent branches of RTG regulation of specific genes in colonies.

## RESULTS

### RTG pathway dysfunction decreases the viability of the specific subpopulation of L cells

**Figure 1:**
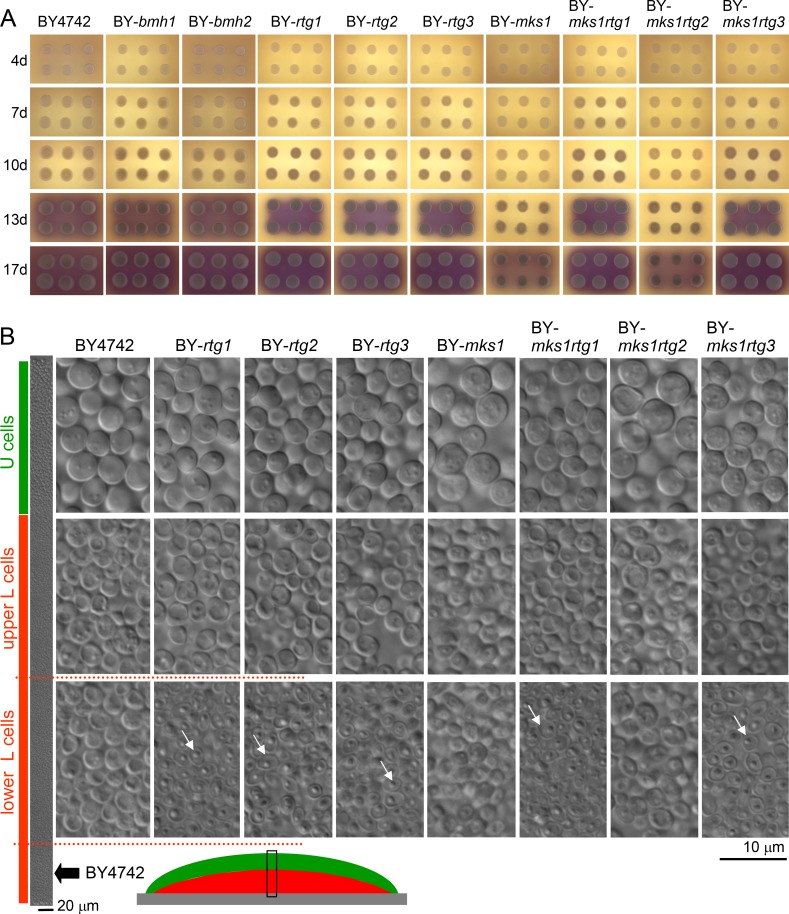
Development and differentiation of yeast colonies formed by wt and KO strains **A.** Colony development and signaling. Colonies were grown on GMA with pH indicator bromcresol purple (yellow at lower pH, turning violet at higher pH, pKa value of ∼ 6.3). Violet indicates the start of the alkali phase. **B.** Morphology of differentiated cells of 17-day-old colonies (in alkali developmental phase) shown on vertical cross-sections of giant colonies. The leftmost panel shows a segment of a cross-section (the position of which is depicted below using a schematic view of a colony cross-section) from a BY4742 colony to show the position of particular cell types in wt colonies. U cells are marked by green, and L cells are marked by red bars. The dotted red line shows the approximate position of the lower L cells, the viability of which is decreased in *rtg*Δ strains. White arrows indicate examples of dead cells.

With the aim of analyzing possible function(s) of the RTG signaling pathway in developing colonies, we prepared a series of strains derived from *Saccharomyces cerevisiae* BY4742 (wt) with individually deleted genes involved in the RTG signaling cascade. We deleted genes for the activators Rtg1p, Rtg2p and Rtg3p and for the major negative regulators identified thus far, i.e., Mks1p, Bmh1p and Bmh2p. Colonies of all knockout (KO) strains (Table 1) were able to pass through the same developmental phases as colonies of the wt strain, although colonies of the BY-*rtg1*, BY-*rtg2* and BY-*rtg3* strains exhibited slightly slower growth than wt colonies, which caused a slight delay in colony entry to the alkali phase (Figure 1A). A more prominent growth delay was evident in BY-*mks1* colonies. The growth of BY-*bmh1* and BY-*bmh2* colonies was similar to that of wt colonies, which is consistent with findings that Bmh1p and Bmh2p can substitute for each other. Preparation of the *BMH1*/*BMH2* double KO strain was unsuccessful, indicating synthetic lethality of the double deletion, as described in some strain backgrounds [28, 29]. Microscopic analysis of cross-sections of 15-day-old giant colonies using Nomarski contrast showed that all colonies differentiated into U and L cells with typical morphological features. However, in contrast to wt colonies, in colonies formed by the BY-*rtg1*, BY-*rtg2* and BY-*rtg3* strains, a large subpopulation of dead shrunk cells (the cells with reduced cellular content) appeared in the lower layers of the L cell population (Figures 1B and S1). These cells were phenotypically similar to the yeast cells undergoing regulated cell death that have been identified in colonies [24]. These data showed that the inability to activate the RTG pathway negatively affects the survival of L cells and that the L cell population is not homogeneous but that smaller subpopulations exist that behave differently in certain aspects.

**Table 1:** Yeast strains used in this study

name	genotype	source
BY4742	*MAT*α, his3Δ1, leu2Δ0, lys2Δ0, ura3Δ0	Euroscarf
BY-*rtg1*	*MAT*α, his3Δ1, leu2Δ0, lys2Δ0, ura3Δ0, rtg1::nat	this study
BY-*rtg2*	*MAT*α, his3Δ1, leu2Δ0, lys2Δ0, ura3Δ0, rtg2::nat	this study
BT-*rtg3*	*MAT*α, his3Δ1, leu2Δ0, lys2Δ0, ura3Δ0, rtg3::nat	this study
BY-*mks1*	*MAT*α, his3Δ1, leu2Δ0, lys2Δ0, ura3Δ0, mks1::nat	this study
BY-*bmh1*	*MAT*α, his3Δ1, leu2Δ0, lys2Δ0, ura3Δ0, bmh1::nat	this study
BY-*bmh2*	*MAT*α, his3Δ1, leu2Δ0, lys2Δ0, ura3Δ0, bmh2::nat	this study
BY-*mks1rtg1*	*MAT*α, his3Δ1, leu2Δ0, lys2Δ0, ura3Δ0, mks1::nat, rtg1::hph	this study
BY-*mks1rtg2*	*MAT*α, his3Δ1, leu2Δ0, lys2Δ0, ura3Δ0, mks1::nat, rtg2::hph	this study
BY-*mks1rtg3*	*MAT*α, his3Δ1, leu2Δ0, lys2Δ0, ura3Δ0, mks1::nat, rtg3::hph	this study
BY-Ato1p-GFP	*MAT*α, his3Δ1, leu2Δ0, lys2Δ0, ura3Δ0, ATO1-yEGFP-nat	this study
BY-Ato2p-GFP	*MAT*α, his3Δ1, leu2Δ0, lys2Δ0, ura3Δ0, ATO2-yEGFP-nat	this study
BY-Ato3p-GFP	*MAT*α, his3Δ1, leu2Δ0, lys2Δ0, ura3Δ0, ATO3-yEGFP-nat	this study
BY-*rtg1*-Ato1p-GFP	*MAT*α, his3Δ1, leu2Δ0, lys2Δ0, ura3Δ0, rtg1::nat, ATO1-yEGFP–kanMX	this study
BY-*rtg1*-Ato2p-GFP	*MAT*α, his3Δ1, leu2Δ0, lys2Δ0, ura3Δ0, rtg1::nat, ATO2-yEGFP–kanMX	this study
BY-*rtg1*-Ato3p-GFP	*MAT*α, his3Δ1, leu2Δ0, lys2Δ0, ura3Δ0, rtg1::nat, ATO3-yEGFP–kanMX	this study
BY-*rtg2*-Ato1p-GFP	*MAT*α, his3Δ1, leu2Δ0, lys2Δ0, ura3Δ0, rtg2::nat, ATO1-yEGFP–kanMX	this study
BY-*rtg2*-Ato2p-GFP	*MAT*α, his3Δ1, leu2Δ0, lys2Δ0, ura3Δ0, rtg2::nat, ATO2-yEGFP–kanMX	this study
BY-*rtg2*-Ato3p-GFP	*MAT*α, his3Δ1, leu2Δ0, lys2Δ0, ura3Δ0, rtg2::nat, ATO3-yEGFP–kanMX	this study
BY-*rtg3*-Ato1p-GFP	*MAT*α, his3Δ1, leu2Δ0, lys2Δ0, ura3Δ0, rtg3::nat, ATO1-yEGFP–kanMX	this study
BY-*rtg3*-Ato2p-GFP	*MAT*α, his3Δ1, leu2Δ0, lys2Δ0, ura3Δ0, rtg3::nat, ATO2-yEGFP–kanMX	this study
BY-*rtg3*-Ato3p-GFP	*MAT*α, his3Δ1, leu2Δ0, lys2Δ0, ura3Δ0, rtg3::nat, ATO3-yEGFP–kanMX	this study
BY-*mks1*-Ato1p-GFP	*MAT*α, his3Δ1, leu2Δ0, lys2Δ0, ura3Δ0, mks1::nat, ATO1-yEGFP–kanMX	this study
BY-*mks1*-Ato2p-GFP	*MAT*α, his3Δ1, leu2Δ0, lys2Δ0, ura3Δ0, mks1::nat, ATO2-yEGFP–kanMX	this study
BY-*mks1*-Ato3p-GFP	*MAT*α, his3Δ1, leu2Δ0, lys2Δ0, ura3Δ0, mks1::nat, ATO3-yEGFP–kanMX	this study
BY-*bmh1*-Ato1p-GFP	*MAT*α, his3Δ1, leu2Δ0, lys2Δ0, ura3Δ0, bmh1::nat, ATO1-yEGFP–kanMX	this study
BY-*bmh1*-Ato2p-GFP	*MAT*α, his3Δ1, leu2Δ0, lys2Δ0, ura3Δ0, bmh1::nat, ATO2-yEGFP–kanMX	this study
BY-*bmh1*-Ato3p-GFP	*MAT*α, his3Δ1, leu2Δ0, lys2Δ0, ura3Δ0, bmh1::nat, ATO3-yEGFP–kanMX	this study
BY-*bmh2*-Ato1p-GFP	*MAT*α, his3Δ1, leu2Δ0, lys2Δ0, ura3Δ0, bmh2::nat, ATO1-yEGFP–kanMX	this study
BY-*bmh2*-Ato2p-GFP	*MAT*α, his3Δ1, leu2Δ0, lys2Δ0, ura3Δ0, bmh2::nat, ATO2-yEGFP–kanMX	this study
BY-*bmh2*-Ato3p-GFP	*MAT*α, his3Δ1, leu2Δ0, lys2Δ0, ura3Δ0, bmh2::nat, ATO3-yEGFP–kanMX	this study
BY-*mks1rtg1*-Ato1p-GFP	*MAT*α, his3Δ1, leu2Δ0, lys2Δ0, ura3Δ0, mks1::nat, rtg1::hph, ATO1-yEGFP-kanMX	this study
BY-*mks1rtg1*-Ato2p-GFP	*MAT*α, his3Δ1, leu2Δ0, lys2Δ0, ura3Δ0, mks1::nat, rtg1::hph, ATO2-yEGFP-kanMX	this study
BY-*mks1rtg1*-Ato3p-GFP	*MAT*α, his3Δ1, leu2Δ0, lys2Δ0, ura3Δ0, mks1::nat, rtg1::hph, ATO3-yEGFP-kanMX	this study
BY-*mks1rtg2*-Ato1p-GFP	*MAT*α, his3Δ1, leu2Δ0, lys2Δ0, ura3Δ0, mks1::nat, rtg2::hph, ATO1-yEGFP-kanMX	this study
BY-*mks1rtg2*-Ato2p-GFP	*MAT*α, his3Δ1, leu2Δ0, lys2Δ0, ura3Δ0, mks1::nat, rtg2::hph, ATO2-yEGFP-kanMX	this study
BY-*mks1rtg2*-Ato3p-GFP	*MAT*α, his3Δ1, leu2Δ0, lys2Δ0, ura3Δ0, mks1::nat, rtg2::hph, ATO3-yEGFP-kanMX	this study
BY-*mks1rtg3*-Ato1p-GFP	*MAT*α, his3Δ1, leu2Δ0, lys2Δ0, ura3Δ0, mks1::nat, rtg3::hph, ATO1-yEGFP-kanMX	this study
BY-*mks1rtg3*-Ato2p-GFP	*MAT*α, his3Δ1, leu2Δ0, lys2Δ0, ura3Δ0, mks1::nat, rtg3::hph, ATO2-yEGFP-kanMX	this study
BY-*mks1rtg3*-Ato3p-GFP	*MAT*α, his3Δ1, leu2Δ0, lys2Δ0, ura3Δ0, mks1::nat, rtg3::hph, ATO3-yEGFP-kanMX	this study
BY-Cit2p-GFP	*MAT*α, his3Δ1, leu2Δ0, lys2Δ0, ura3Δ0, CIT2-yEGFP-kanMX	this study
BY-*rtg1*-Cit2p-GFP	*MAT*α, his3Δ1, leu2Δ0, lys2Δ0, ura3Δ0, rtg1::nat, CIT2-yEGFP-kanMX	this study
BY-*rtg2*-Cit2p-GFP	*MAT*α, his3Δ1, leu2Δ0, lys2Δ0, ura3Δ0, rtg2::nat, CIT2-yEGFP-kanMX	this study
BY-*rtg3*-Cit2p-GFP	*MAT*α, his3Δ1, leu2Δ0, lys2Δ0, ura3Δ0, rtg3::nat, CIT2-yEGFP-kanMX	this study
BY-*mks1*-Cit2p-GFP	*MAT*α, his3Δ1, leu2Δ0, lys2Δ0, ura3Δ0, mks1::nat, CIT2-yEGFP-kanMX	this study
BY-*bmh1*-Cit2p-GFP	*MAT*α, his3Δ1, leu2Δ0, lys2Δ0, ura3Δ0, bmh1::nat, CIT2-yEGFP-kanMX	this study
BY-*bmh2*-Cit2p-GFP	*MAT*α, his3Δ1, leu2Δ0, lys2Δ0, ura3Δ0, bmh2::nat, CIT2-yEGFP-kanMX	this study
BY-*mks1rtg1*-Cit2p-GFP	*MAT*α, his3Δ1, leu2Δ0, lys2Δ0, ura3Δ0, mks1::nat, rtg1::hph, CIT2-yEGFP-kanMX	this study
BY-*mks1rtg2*-Cit2p-GFP	*MAT*α, his3Δ1, leu2Δ0, lys2Δ0, ura3Δ0, mks1::nat, rtg1::hph, CIT2-yEGFP-kanMX	this study
BY-*mks1rtg3*-Cit2p-GFP	*MAT*α, his3Δ1, leu2Δ0, lys2Δ0, ura3Δ0, mks1::nat, rtg1::hph, CIT2-yEGFP-kanMX	this study
BY-Rtg1p-GFP	*MAT*α, his3Δ1, leu2Δ0, lys2Δ0, ura3Δ0, RTG1-yEGFP-kanMX	this study
BY-Rtg2p-GFP	*MAT*α, his3Δ1, leu2Δ0, lys2Δ0, ura3Δ0, RTG2-yEGFP-kanMX	this study

### Rtg proteins and Mks1p regulate Cit2p production specifically in upper layers of L cells

**Figure 2:**
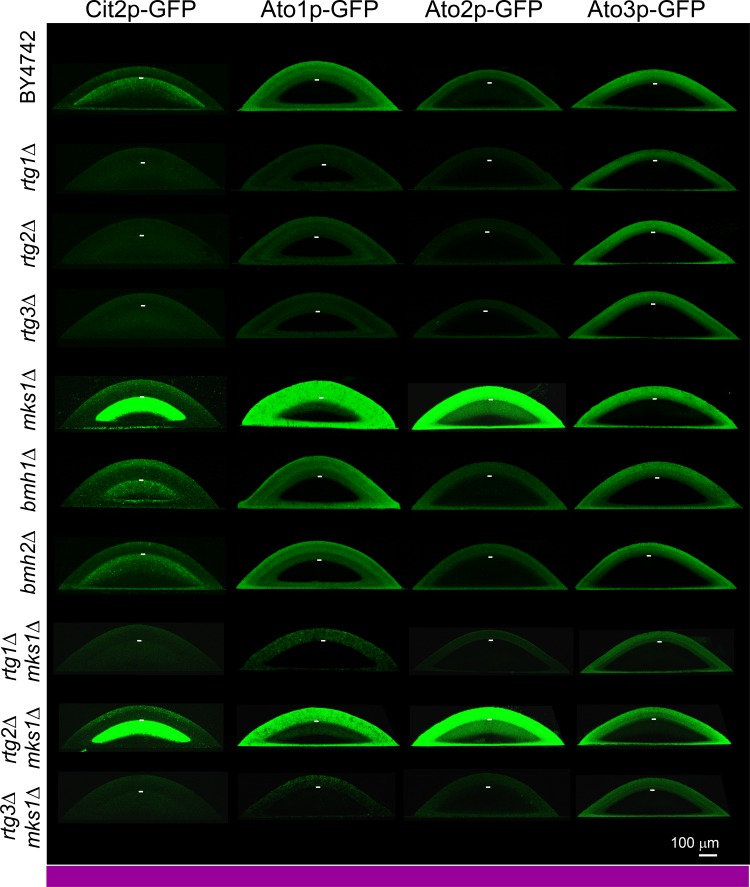
Production of Cit2p, Ato1p, Ato2p and Ato3p fused with GFP within differentiated colonies of wt and KO strains occurring in alkali phase Localization of the indicated protein is visualized as green fluorescence on vertical colony cross-sections of 4-day-old microcolonies (occurring in full alkali developmental phase, as marked by the violet bar) by 2PE-CM. The same confocal microscopy setup was used for all colonies (wt and KO) producing the indicated protein. Thus, the intensity of the fluorescence roughly reflects changes in the level of the indicated protein in colonies of different strains. Approximate position of the border between U and L cells in individual colonies is indicated by the short white lines.

The gene *CIT2* is one of the typical targets of the RTG pathway described thus far*.* Our aim was to analyze the potential expression of *CIT2* directly in cell subpopulations of developing colonies. Therefore, we prepared strains containing the genomic *CIT2* gene fused with the gene encoding GFP, thus producing the Cit2p-GFP protein tagged on its C terminus, which was derived from the above-described wt and KO strains (Table 1). Colonies of all constructed strains were first analyzed in terms of their morphology, differentiation and alkalization, demonstrating that *CIT2* fusion with GFP did not affect colony development (data not shown). Alkali-phase microcolonies (4-day-old) and giant colonies (17-day-old) of constructed strains were then analyzed by two-photon excitation confocal microscopy (2PE-CM) and wide-field microscopy, respectively. Cit2p-GFP was detected in cell subpopulations localized to the upper layers of L cells of both colony types, i.e., to the subpopulation that differed from the dying L cell subpopulation present in colonies of strains with deleted *RTG* genes (Figures 2 and S2). Cit2p-GFP production in any of the colony subpopulations was fully dependent on the presence of any of the three *RTG* genes. The absence of Mks1p (but not the individual absence of Bmh1p or Bmh2p) dramatically increased the Cit2p level, specifically in the upper layers of L cells of the microcolonies, while the effect of *mks1*Δ on the Cit2p-GFP level in the upper L cells of chronologically older giant colonies was negligible (data not shown). The absence of Mks1p did not cause any Cit2p production in either U cells or lower layers of L cells of both colony types.

**Figure 3:**
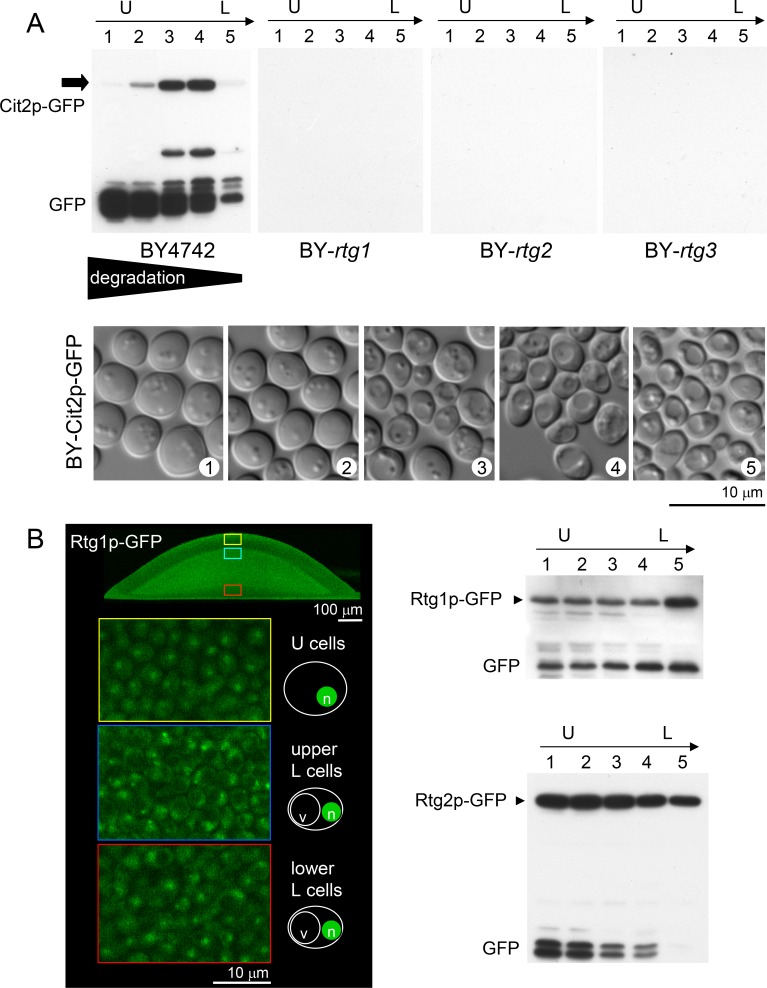
Comparison of amounts of Cit2p-GFP, Rtg1p-GFP and Rtg2p-GFP in different subpopulations of giant colonies **A.** WB analysis of the amount of Cit2p-GFP in 5 cell layers (1-5) separated from 14-day-old alkali phase colonies formed by wt and *rtg*Δ strains, starting from the top of the colony (i.e., from upper U cells, layer 1) and ending at the lowest layers of L cells near the agar. The lower panel shows the morphology of cells from layers 1-5 separated from wt colonies producing Cit2p-GFP that were used for WB analysis. **B.** Left panel, localization of Rtg1p-GFP in cells from microcolonies (4-day-old) of colony cross-sections as determined by 2PE-CM. Nuclear localization in U cells and in both types of L cells is shown on magnified images marked in color in colony cross-sections. Right panels, comparison of Rtg1p-GFP and Rtg2p-GFP amounts in 5 cell layers (1-5 as in A) of 14-day-old alkali-phase wt colonies by WB. Loading controls are in Figure S5.

To obtain more precise Cit2p quantification in particular subpopulations, we separated 5 layers of cells from differentiated giant colonies of the wt and KO strains and compared the Cit2p-GFP levels by Western blot (WB) analysis (Figure 3A). Consistent with the microscopy results, the highest levels of Cit2p-GFP were found in the upper layers of L cells and in cells at the border between the U and L cells. U cells contained high levels of free GFP, indicating efficient Cit2p-GFP degradation most likely by autophagy, which is active in U cells [25]. The amount of degradation decreased toward the lower layers of colonial cells. No GFP fluorescence was detected in the lower layers of L cells (Figure 2), which, together with the low signal of Cit2p-GFP and free GFP in WBs of separated cells (Figure 3A), confirmed that Cit2p is not synthesized in these cells. The borders between WB fractions were less sharp than the borders observed by microscopy because of slight cross-contamination of the fractions of separated cell layers (Figure 3A). In addition, the low amount of free GFP detected in fraction 5 could also partly originate from the thin layer (of one to three cells) of cells resembling U cells always visible at the bottom of the colony [25, 30].

### Rtg proteins and Mks1p regulate Ato1p and Ato2p production specifically in U cells

**Figure 4:**
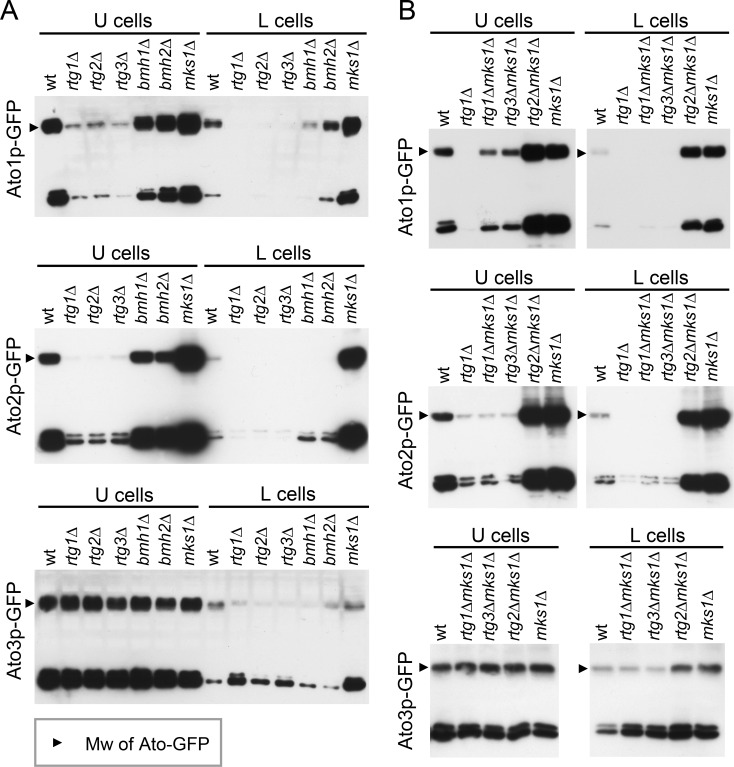
Comparison of amounts of Ato proteins in U and L cells from giant colonies of wt and KO strains Levels of Ato1p-, Ato2p- and Ato3p-GFP in U and L cells separated from 14-day-old giant colonies formed by wt and KO strains were determined by WB. Ato-GFP, upper band; GFP, a product of Ato-GFP degradation, lower band. **A.** Comparison of wt colonies and colonies of strains with individual deletion of regulators involved in the RTG pathway. **B.** Comparison of wt colonies and colonies of strains with double deletions of *RTG* and *MKS1* genes. Loading controls are in Figure S5.

Ato proteins are prominent markers of U cells [25, 30]. When screening for *ATO* gene expression in the absence of different transcription regulators (unpublished data), we identified a positive effect of Rtg proteins on *ATO1* expression. Therefore, we prepared a series of strains individually producing Ato1p-GFP, Ato2p-GFP or Ato3p-GFP derived from *RTG* gene KO strains (Table 1). C-terminal tagging of *ATO* genes does not affect the functions of their encoded proteins [31], as confirmed by analyzing the colonies of all constructed strains (data not shown). Microscopic analysis of Ato-GFP level in situ within alkali-phase microcolonies and giant colonies showed that Ato1p-GFP and Ato2p-GFP production in U cells depends on the function of all three *RTG* genes, while Ato3p-GFP production occurs normally in colonies of KO strains with any *RTG* gene deletion (Figures 2 and S3). The absence of Mks1p increased the levels of Ato1p-GFP and Ato2p-GFP not only in U cells of both colony types but also in upper layers of L cells, i.e., in the layers where Cit2p-GFP is typically produced. The precise quantification of Ato-GFP proteins in separated U and L cell subpopulations by WB analysis fully confirmed the differences observed by microscopy (Figure 4A). The WBs also showed that in contrast to Cit2p production, which was completely abolished in the absence of any of the Rtg proteins, basal levels of Ato1p-GFP and Ato2p-GFP remained present in U cells from *rtg*Δ colonies. Interestingly, the absence of the repressor Mks1p also slightly increased the Ato3p-GFP level in L cells (Figure 4A). The *ATO1* and *ATO2* mRNA levels in microcolonies of wt and KO strains (Figure S4A) differed consistently with the effect of the presence/absence of these regulators on Ato1p-GFP and Ato2p-GFP protein amounts. This result confirmed that Rtg/Mks1p regulate *ATO1* and *ATO2* expression in colonies at the transcriptional level.

### The repressor Mks1p functions dependent and independent of the RTG pathway

The above data showed that the repressor Mks1p affects different gene targets in different cell subpopulations. To determine whether Mks1p affects the expression of these genes strictly *via* repression of the RTG pathway, we prepared an additional set of strains derived from the BY-*mks1*-Cit2p-GFP, BY-*mks1*-Ato1p-GFP, BY-*mks1*-Ato2p-GFP and BY-*mks1*-Ato3p-GFP strains, each of which had an additional deletion of one of the three *RTG* genes (Table 1). Then, the levels of Cit2p-GFP and Ato-GFP proteins in individual colony subpopulations were analyzed in situ within microcolonies by 2PE-CM. The deletion of either the *RTG1* or *RTG3* gene in BY-*mks1*-Cit2p-GFP eliminated Cit2p-GFP from the colonies. In contrast, the deletion of *RTG2* had no effect, as the Cit2p-GFP level was comparable with the Cit2p-GFP level in the BY-*mks1*-Cit2p-GFP strain (Figure 2). Thus, *CIT2* expression in colonies is fully under the control of the Mks1p-RTG pathway and is directed through signaling from mitochondria *via* Rtg2p.

The deletion of the *RTG1* or *RTG3* gene in *mks1*Δ strains eliminated the production of Ato1p-GFP and Ato2p-GFP in the upper layers of L cells and significantly diminished the production of both proteins in U cells (Figures 2 and 4B). However, the levels of Ato1p-GFP in the U cells of *mks1*Δ/*rtg1*Δ and *mks1*Δ/*rtg3*Δ colonies remained slightly but significantly higher than the levels of Ato1p-GFP in the U cells of *rtg1*Δ and *rtg3*Δ colonies as shown by WBs (Figure 4B). The deletion of the *RTG2* gene did not affect the high Ato1p-GFP and Ato2p-GFP levels typical of *mks1*Δ colonies or the slightly increased Ato3p-GFP level in upper L cells of BY-*mks1*-Ato3p-GFP colonies (Figures 2 and 4B). These data demonstrated that the Mks1p-RTG pathway is the major pathway that activates Ato1p and Ato2p production in U cells *via* Rtg2p-mediated signaling from mitochondria. In addition, these data showed that Mks1p partially represses the *ATO1* gene in U cells, independent of the RTG pathway.

### Rtg proteins and Mks1p regulate the viability of lower L cells

Neither Cit2p-GFP nor Ato-GFP production nor any *mks1*Δ effect on the production of these proteins was observed in the lower layers of L cells, the viability of which was significantly decreased in the absence of any of the *RTG* genes. This finding implied that another branch of the RTG pathway is active in these cells and regulates other currently unknown target(s). To determine whether the Rtg1p-Rtg3p transcription regulator localizes to the nuclei of these cells and the level of Rtg2p, we prepared strains producing Rtg1p or Rtg2p labeled with GFP. Then, we analyzed Rtg1p-GFP cellular localization in situ within the colonies by 2PE-CM and Rtg1p-GFP and Rtg2p-GFP levels in different cell subpopulations separated from the colonies by WBs. Figure 3B shows that Rtg1p-GFP is present in the nuclei of all colonial cells, including lower L cells, and that a relatively high level of Rtg2p is present in all cell subpopulations.

The deletion of *MKS1*, which possibly results in hyperactivation of the RTG pathway at the beginning of colony development, caused a partial growth defect in colonies. This defect was reversed by additional deletion of *RTG1* or *RTG3*, but not of *RTG2* (Figure 1A), which confirmed that premature RTG signaling is responsible for slower colony growth and not for other RTG-independent Mks1p function(s). In contrast, the subpopulation of dead L cells was not present in colonies of the BY-*mks1* and BY-*mks1rtg2* strains with a hyperactive RTG pathway. However, this dead cell subpopulation was found in the BY-*mks1rtg1* and BY-*mks1rtg3* strains, similar to that of colonies of strains with deletions of any single *RTG* gene (Figure 1B). Hence, premature hyperactivation of the RTG pathway in young colonies not yet reaching starvation negatively affects the subsequent overall growth of colonies, whereas the inability to activate the RTG pathway later during colony aging (most likely when conditions become worse) causes this specific L cell subpopulation to die.

### RTG signaling in acidic-phase colonies

Mks1p-Rtg regulation of different gene targets in the U and L cells of differentiated colonies during the alkali phase is shown above. Next, we determined whether (and, if so, how) *RTG* genes and the repressor Mks1p are involved in the regulation of *CIT2* and *ATO* genes in acidic-phase colonies, i.e., before the formation of fully developed U and L cells. Hence, we analyzed acidic-phase microcolonies of the above-described wt and KO strains by 2PE-CM.

**Figure 5:**
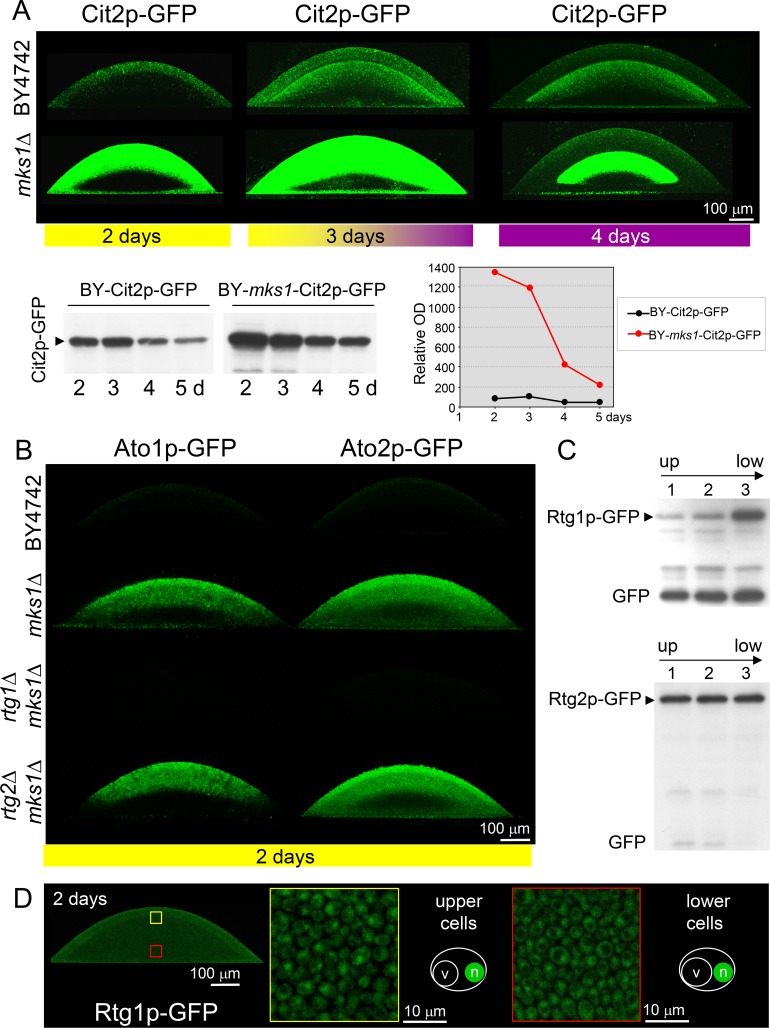
Effect of developmental phase on the production and localization of Ato1p, Ato2p, Cit2p, Rtg1p and Rtg2p in cells of colonies of different strains **A.** Levels and localization of Cit2p-GFP within colonies occurring in acidic phase (left, yellow bar), immediately upon entering the alkali phase (center) and during the alkali phase (right, violet bar). Changes in the Cit2p-GFP levels in whole microcolonies of wt and *mks1*Δ strains during the interval of 2-5 days are shown below by WB analysis (left) and are quantified in the graph (right). **B.** Ato1p-GFP and Ato2p-GFP levels and localization within 2-day-old microcolonies occurring in acidic phase (marked by a yellow bar below the image) as determined by 2PE-CM. **C.** Amounts of Rtg1p-GFP and Rtg2p-GFP in three layers (starting from the top of the colony, layer 1) separated from 6-day-old acidic-phase giant colonies. **D.** Localization of Rtg1p-GFP in cells from 2-day-old microcolonies as determined by 2PE-CM. Up, upper part of the colony; low, lower part of the colony. Loading controls (for A and C) are in Figure S5.

Cit2p-GFP is produced in the upper cell layers of 2-day-old acidic-phase colonies, and this production is partially repressed by Mks1p, as the Cit2p-GFP level was substantially increased in colonies of the BY-*mks1*-Cit2p-GFP strain (Figure 5A). Monitoring the level and localization of Cit2p-GFP during the transition of microcolonies from the acidic to the fully alkali phase (interval from 2 to 4 days of microcolony development) (Figure 5A) showed that Cit2p-GFP is present in both the upper cell layers and the upper layers of lower cells in the early alkali phase and that the expression level in both regions is partially repressed by Mks1p. WB quantification showed that the repressive effect of Mks1p on *CIT2* expression gradually decreased during the interval from the third to the fifth day of microcolony development (i.e., during the aging of alkali-phase microcolonies). This finding is consistent with the observed negligible effect of *MKS1* deletion on Cit2p-GFP production in L cells of 15-day-old alkali-phase giant colonies.

Consistent with previous results [20, 32], Ato1p-GFP and Ato2p-GFP proteins were not produced in 2-day-old acidic phase microcolonies. However, the level of these proteins was greatly increased in the absence of Mks1p. The positive effect of *mks1*Δ on Ato1p-GFP/Ato2p-GFP production was strictly dependent on the presence of functional *RTG1*/*RTG3* genes and independent of the *RTG2* gene (Figure 5B). No residual Ato1p-GFP or Ato2p-GFP fluorescence was detectable in the absence of *RTG1*/*RTG3* genes in a *mks1*Δ background, which showed that Mks1p is the only repressor of *ATO1* and *ATO2* genes in the upper cell layers of acidic-phase colonies.

As in the alkali phase, Rtg1p-GFP and Rtg2p-GFP were present in all three cell subpopulations separated from acidic-phase giant colonies (Figure 5C) and Rtg1p-GFP localized to nuclei in all cells in these colonies (Figure 5D). These findings indicate additional roles of the RTG pathway in cell subpopulations where Cit2p is not produced.

## DISCUSSION

**Figure 6:**
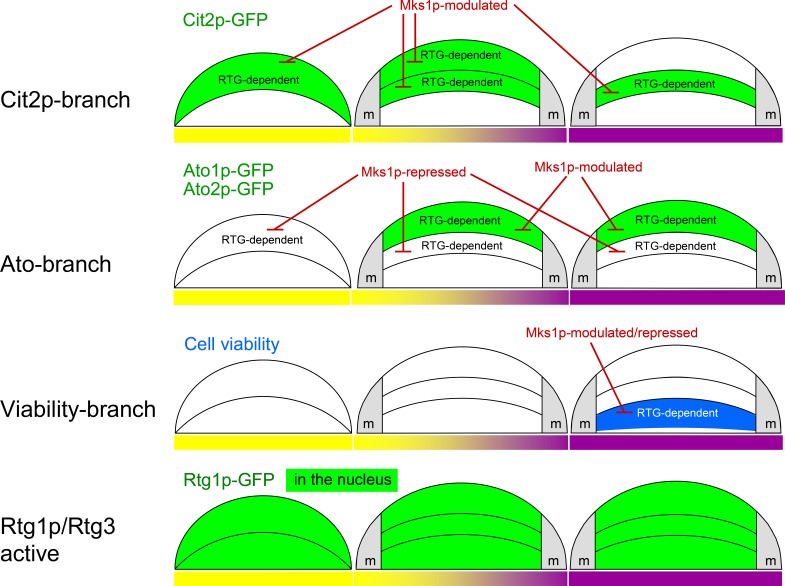
Summary of RTG regulation in developing colonies Diagrammatic illustrations of the functions of proteins of three branches of the RTG pathway in cell subpopulations localized in different layers of colonies occurring in late acidic (left, yellow bar), early alkali (middle, yellow/violet bar) and fully alkali (right, violet bar) colonies. The repressing or modulating effect of Mks1p on Cit2p and Ato1p/Ato2p production is indicated. m, margin regions of colonies.

**Figure 7:**
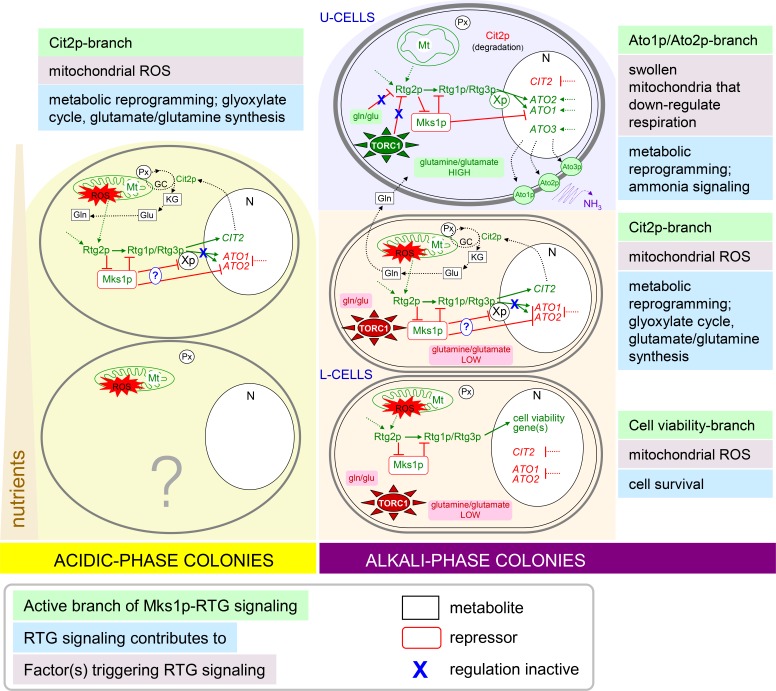
Schematic model of relations among newly identified branches of RTG regulation and other metabolic and regulatory pathways of U and L cells and cells within acidic-phase colonies Solid green arrows/red lines indicate activation/repression by a known regulator. Dotted green arrows/red lines indicate activation/repression by not yet identified regulator. Dotted black arrows indicate a metabolic process or transport. Genes, proteins, processes or metabolites marked in green are induced/high and those in red are repressed/low in the particular cell type. Expression of *ATO1*/*ATO2* and *CIT2* is repressed and/or activated in particular subpopulations also by additional unknown regulatory proteins. Xp, not yet identified factor co-regulating *ATO1*/*ATO2* expression together with Rtg1p/Rtg3p; Mt, mitochondrion; Px, peroxisomes; N, nucleus; gln, glutamine; glu, glutamate; KG, α-ketoglutarate; GC, glyoxylate cycle.

New data revealed that signaling from mitochondria *via* the RTG pathway is crucial in certain stages of the development and differentiation of yeast colonies. The inactivation of this signaling caused lethality to the specific cell subpopulation localized to lower layers of L cells and diminished the production of specific proteins in both U cells (Ato1p and Ato2p) and the upper layers of L cells (Cit2p) as well as in potential ancestors of these cells in acidic-phase colonies (Figure 6). Hence, signaling from mitochondria *via* the RTG pathway plays a role in all subpopulations of fully differentiated colonies and in some of their progenitors; however, the roles are different in particular subpopulations and result in different cell fates. As mitochondria with differently affected functionality are present in U *versus* L cells, the particular functional state of mitochondria could stimulate different targeting of RTG signaling in the three colonial cell subpopulations. The relation of the newly identified branches of RTG regulation to other pathways described previously in U and L cells and to the metabolic properties of these cell types are discussed below and summarized in model scheme (Figure 7).

All three positive activators, Rtg1p, Rtg2p and Rtg3p, as well as the repressor Mks1p are important in every type of mitochondrial signaling in the colonies described in this paper. However, these regulators regulate different gene targets in specific cell subpopulations differently. In U cells, two of the three Ato proteins (the prominent markers of U cells, the production of which is sharply induced when colonies enter alkali phase, [20, 31]) are under major control of RTG signaling, including modulation of the expression of *ATO1* and *ATO2* genes by the repressor Mks1p. This modulation means that the removal of this repressor further enhances the considerable *ATO1* and *ATO2* gene expression levels characteristic of wt U cells. In the acidic phase progenitors of U cells, *ATO1*/*ATO2* expression is completely repressed by Mks1p. In contrast, the expression of the third *ATO* gene *ATO3* is almost unaffected by the RTG pathway in colonies. Because *ATO3* was identified during studies of respiratory deficient yeast strains in liquid cultures as a gene regulated by another uncharacterized mitochondrial signaling pathway [33], *ATO3* expression in colonies could also be regulated by signaling from mitochondria *via* another retrograde pathway independent of the *RTG* genes.

Although RTG signaling is active in U cells, as shown by *ATO1*/*ATO2* expression, the typical RTG pathway target Cit2p is present in upper cells only in acidic-phase colonies and in the early stages of U/L colony differentiation (early alkali phase). Later, in fully differentiated U cells, Cit2p is effectively degraded, and its new synthesis is stopped by an unknown mechanism independent of the repressor Mks1p because deletion of the *MKS1* gene did not increase Cit2p synthesis. At this developmental period, only the upper layers of L cells continue to synthesize the Cit2p protein.

The L cell population is composed of at least two cell subpopulations that exhibit active RTG signaling: the subpopulation in the upper layers of L cells, in which Cit2p production is active, and the subpopulation in the lower L layers, in which the functionality of the RTG pathway is indispensable for cell viability (but not for the production of any of the three RTG-regulated proteins described here). All three *RTG* proteins and the repressor Mks1p are involved in both types of regulation in L cells.

The differences in gene targets regulated by the Mks1p/Rtg pathway in specific cell subpopulations suggest the presence of additional regulators responsible for the diversification of different branches of mitochondrial RTG signaling. For example, Mks1p is functional in both Cit2p expression regions and Ato expression regions because its deletion enhances the production of particular proteins. However, *ATO* gene expression in Cit2p regions is fully blocked by Mks1p, which suggests that Mks1p can either directly repress *ATO1* and *ATO2* (and possibly other genes) or can repress additional currently unknown co-activator(s) of these genes, in addition to its typical negative regulation of the RTG pathway. This potential Mks1p-repressed co-activator can activate its gene targets only under the conditions of active Rtg1p-Rtg3p (as deletion of *RTG1*/*RTG3* suppresses the *mks1*Δ effect) and thus could contribute to the divergent RTG regulation of specific genes in specific cell subpopulations (Figure 7). In contrast, the expression of *CIT2* in Ato regions is blocked independent of Mks1p (as *mks1*Δ has no effect), which suggests either the presence of an additional *CIT2* repressor or the absence of any putative *CIT2*-specific co-activator of Rtg1p-Rtg3p.

The RTG pathway is activated in U cells by Rtg2p, which most likely detects a signal from dampened ROS-free mitochondria, independent of the fact that U cells are metabolically active cells that contain high levels of glutamine/glutamate and have an active TORC1 pathway [22, 25], which both have been shown to inhibit the RTG pathway in yeast cells in liquid cultures [10-12]. This finding supplements other unusual features of U cells described previously [22, 25], most of which are paralleled in tumor cells of mammals, such as aerobic glycolysis, glutaminolysis, ammonia production and autophagy [27, 34]. The finding that the RTG pathway targets in U cells differ from those activated by the same pathway in starving cells argues for the existence of other signaling branches driven by differently affected mitochondria, which use the same core gene products Rtg/Mks1p but are specialized according to the particular cell type and likely include different additional regulatory elements. This conclusion is supported by the fact that even starving cells such as L cells can differ in terms of the functions of mitochondrial RTG signaling. In one subpopulation, active signaling is represented by *CIT2* expression, and in the second subpopulation, RTG gene functionality is indispensable for cell viability by a yet unknown mechanism. The active RTG pathway in L cells that harbor low levels of glutamate, as well as particularly low levels of glutamine and inactive TORC1, is consistent with the generally accepted model of RTG pathway regulation. In L cells with higher levels of ROS and a starvation phenotype, mitochondria most likely decrease functionality to a level that induces an RTG response, although L cells are capable of respiration when separated from colonies. No direct information regarding mitochondrial respiratory status in situ within colonies is available and, in fact, is difficult to obtain.

RTG signaling is related to metabolic properties of the cell subpopulations and to their interactions during colony development. In yeast, the RTG pathway contributes to changes in the TCA cycle and activation of peroxisomal fatty acid β-oxidation and glyoxylate shunt, resulting in the increased synthesis of glutamate, a precursor of most amino acids [13]. Cit2p is a typical marker protein linked to these changes [35]. Cells localized to the upper regions of younger acidic-phase colonies (i.e., U cell progenitors) produce Cit2p, and a significant Cit2p level persists in newly formed U cells in the early stages of U/L cell differentiation. Hence, the RTG pathway could be involved in the synthesis of glutamate/glutamine before the full differentiation of cells in upper colony layers to typical U cells. After colony differentiation, the Cit2p protein is present predominantly in the upper layers of L cells, i.e., in cells close to the U cells. Previous findings suggested that L cells synthesize and release certain nutrients, including glutamine, to feed U cells [25]. Therefore, we can hypothesize that the preservation of the Cit2p-related branch of RTG signaling, particularly in upper layers of L cells, contributes to such feeding, during which glutamine and glutamate synthesized and released from upper L cells are immediately consumed by U cells. Findings of much higher levels of glutamine/glutamate in U cells compared with L cells [25] support this hypothesis. The second branch of RTG signaling is active in fully differentiated U cells. This branch, most likely with the help of additional co-activators, fulfills other important regulatory tasks independent of Cit2p and related processes that include the sharp induction of *ATO1*/*ATO2* genes, whose products are involved in ammonia signaling; this signaling is crucial for the metabolic reprogramming of U cells and for the differentiation of colonies [20, 24, 25, 36]. Ammonia production is one of the typical features shared by U cells and tumor cells. The third branch of RTG signaling (not accompanied by either Cit2p or Ato production) is related to the viability of the lower L cell subpopulation, i.e., to another fraction of L cells that most likely provide nutrients to U cells. These cells are localized farther from the U cells but closer to the nutritive agar, which could make them important players in nutrient cycling. The observed cell death phenotype of these cells progressed during colony aging, being more prominent in older giant colonies than in faster developing microcolonies (data not shown). This observation is consistent with previous findings that, in contrast to typical features of U cells that are linked to the switching of colonies to alkali phase and independent of the absolute age of colonies, stress- and aging-related features (including a gradual decrease in intracellular amino acids) are more prominent in chronologically older L cells of giant colonies existing longer in the neighborhood of U cells than in the younger L cells of microcolonies [22]. Hence, these new data suggest the intriguing possibility that mitochondrial retrograde signaling is involved in the survival of cells that gradually provide nutrients to other cells and that develop features resembling the cachexia phenotype of mammalian tissues [37] during the aging of differentiated colonies. The clear separation of the Cit2p-related branch of RTG signaling from the viability-related branch also suggests that the positive effect of the RTG pathway on yeast survival in liquid cultures [38] could be less dependent on Cit2p-related metabolic reprogramming than originally supposed.

In conclusion, our novel findings show that the roles of RTG signaling in yeast are far more complex, affect more cellular processes than hitherto anticipated, and involve a number of yet unknown regulatory elements. Divergent branches of active RTG signaling were observed in parallel at the same time-point of colony development; these branches were specific to particular cell-types within differentiated colonies. Other responses appeared at other time-points of colony differentiation. The observed RTG signaling behaviors are triggered by distinct mitochondrial alterations that differ significantly for U, L and other colonial cell types. Multiple mitochondrial alterations most likely reflect complex mitochondrial dynamics related to the metabolic differences among differentiated cell types. The described heterogeneity of RTG signaling in yeast colonies closely resembles the pleiotropic mitochondrial retrograde signaling in mammals, which includes a number of parallel regulatory events occurring under different conditions and in different cells; this signaling is regulated through a variety of largely unknown factors. For example, the retrograde signaling that involves the transcriptional regulator Myc (the protein overexpressed in over 70% of all human cancers and a structural homolog of Rtg1p, [15]) can affect a variety of processes, such as the cell cycle, cell death, cell adhesion and cellular metabolism, including glutaminolysis and metabolic pathways that provide precursors for biosynthetic reactions [39]. Hence, mitochondrial retrograde signaling is a universal mechanism in eukaryotes from yeast to mammals, and its activation is connected with many cellular metabolic processes, including those leading to pathophysiological changes such as tumorigenesis. The finding of additional regulatory branches, targets and regulatory elements of mitochondrial retrograde signaling using yeast colonies that metabolically resemble tumor-affected organisms thus suggests prospects for the identification of similar elements of retrograde signaling in mammals.

## MATERIALS AND METHODS

### Yeast strains and cultivation

The wt *S. cerevisiae* strain BY4742 (*MATa*, *his3*Δ, *leu2*Δ, *lys2*Δ, *ura3*Δ) was used. The strains with gene deletions and C-terminal GFP fusions (Table 1) used in this study were derived from BY4742 according to [40] and [41] by transforming the cells with DNA cassettes generated by PCR. C-terminal GFP fusion strains were constructed using either GFP-KanMX or GFP-nat integrative cassette amplified by PCR from plasmid pKT127 or pOH11, respectively. Knockout strains were prepared using plasmids pUG6+25 (nat^r^), pUG6 (kan^r^) and pUG6+32 (hph^r^) (obtained from EUROSCARF collection). Yeast cells were transformed as described in [42].

Giant yeast colonies were grown (six per plate) at 28°C on GMA (1% yeast extract, 3% glycerol, 1% ethanol, 2% agar, and 10 mM CaCl_2_, with or without the pH indicator bromocresol purple). Microcolonies were grown at an approximate density of 5 × 10^3^ per plate.

### Colony imaging to monitor growth and development

Colony images were captured in incident and/or transmitted light. A ProgRes^®^ CT3 CMOS camera with a Navitar objective and the NIS Elements software (Laboratory Imaging) were used.

### Separation of cells from differentiated colonies and determination of protein amounts by WBs

Particular cell layers, three from acidic phase colonies and five or two (U and L cells) from alkali phase colonies were stepwise harvested from colonies by micromanipulation. Presence of individual cell-types (and the purity of the fractions) was controlled by microscopy using Nomarski contrast as shown in Figure 3A (for five cell layers). The detection of GFP tagged proteins in the fractions by Western blots was performed as described previously [32]. In brief, cells were broken by glass beads, and proteins of cell lysates were subjected to SDS-PAGE. After the proteins were transferred to a PVDF membrane, GFP was detected by mouse monoclonal horseradish peroxidase (HRP)-conjugated anti-GFP antibody (Santa Cruz). The peroxidase signal was visualized using Super Signal West Pico (Pierce) on Super RX medical X-ray film (Fuji). The levels of the individual proteins were evaluated by UltraQuant 6.0. To minimize the effect of band saturation, less exposed WBs were usually used for quantification; the quantification of the most concentrated samples could be partially affected by their saturation. Membranes stained by Commassie blue were used as loading controls (Figure S5).

### RNA isolation and northern blotting

Microcolonies (2- and 5-day-old) were collected and suspended in TES buffer (10 mM Tris, pH 7.5, 10 mM EDTA, 0.5% SDS). Total RNA was isolated using the hot phenol method [20]. The total RNA concentration was quantified with a spectrophotometer (Unicam Helios Gamma). Fifteen micrograms of total RNA were denatured in loading buffer with formamide, separated in 1.5% agarose gel and then transferred to a positively charged nylon membrane (Amersham Hybond^TM^-XL, GE Healthcare Ltd). The membranes were hybridized with specific DNA probes prepared using a random primer labeling kit (Takara)*.* The PCR fragments of *ATO1, ATO2* and *CIT2* were labeled with [α-^32^P] dCTP. The rRNA content was visualized by ethidium bromide staining and used as a loading control (Figure S4B).

### Microscopic analysis of cells within the colony structure

Microcolonies were visualized by 2PE-CM according to [30]. In brief, colonies were embedded in low-gelling agarose and cut vertically down the middle. The cut surface was placed on a coverslip, and colony side views were obtained by 2P-CM. A true confocal scanning microscope (SP2 AOBS MP; Leica) was used, fitted with a mode-locked laser (Ti:Sapphire Chameleon Ultra; Coherent Inc.) for two-photon excitation and 20×/0.70 and 63×/1.20 water immersion plan Apochromat objectives. An excitation wavelength of 920 nm was used, and the GFP fluorescence signal was recorded in non-descanned mode, in which the light was collected directly behind the objective by the external PMT4 detector after passing through an E700SP short pass filter (F1), followed by a 525DF25 band pass filter (F2). Images of microcolonies older than 2 days were composed of two stitched fields of view.

The visualization of giant colony cross-sections was performed according to [25]. In brief, intact colonies were embedded in 2% agarose gel and sectioned using a Leica VT1200S vibrating microtome. Sections (20 μm) from central colony parts or individual cells separated from colonies by micromanipulation were examined using Leica DMR and Carl Zeiss Axio Observer.Z1 fluorescence microscopes with a GFP filter or DIC. DIC visualization was used for determination of presence of shrunk dead cells lacking most of the intracellular material as shown previously [24].

## SUPPLEMENTARY FIGURES










