# Supplementary material for: Divergent branches of mitochondrial signaling regulate specific genes and the viability of specialized cell types of differentiated yeast colonies
Source: Oncotarget. 2016 Mar 15;7(13):15299–314. doi: 10.18632/oncotarget.8084 (PMC4941242; doi:10.18632/oncotarget.8084)
Supplement: Supplementary file 1 [file oncotarget-07-15299-s001.pdf]

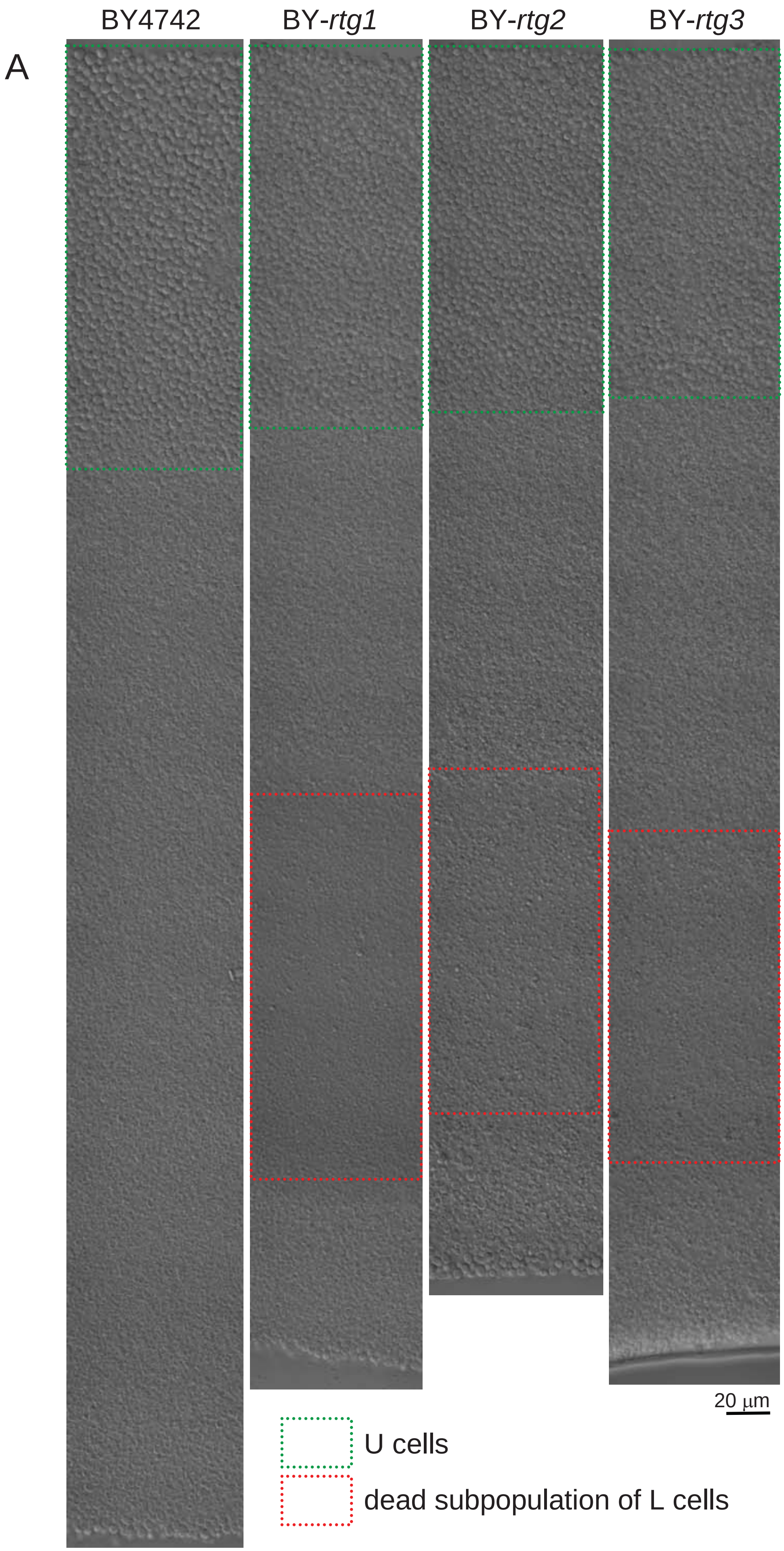

A
BY4742
BY-*rtg1*
BY-*rtg2*
BY-*rtg3*
20 μm
U cells
dead subpopulation of L cells

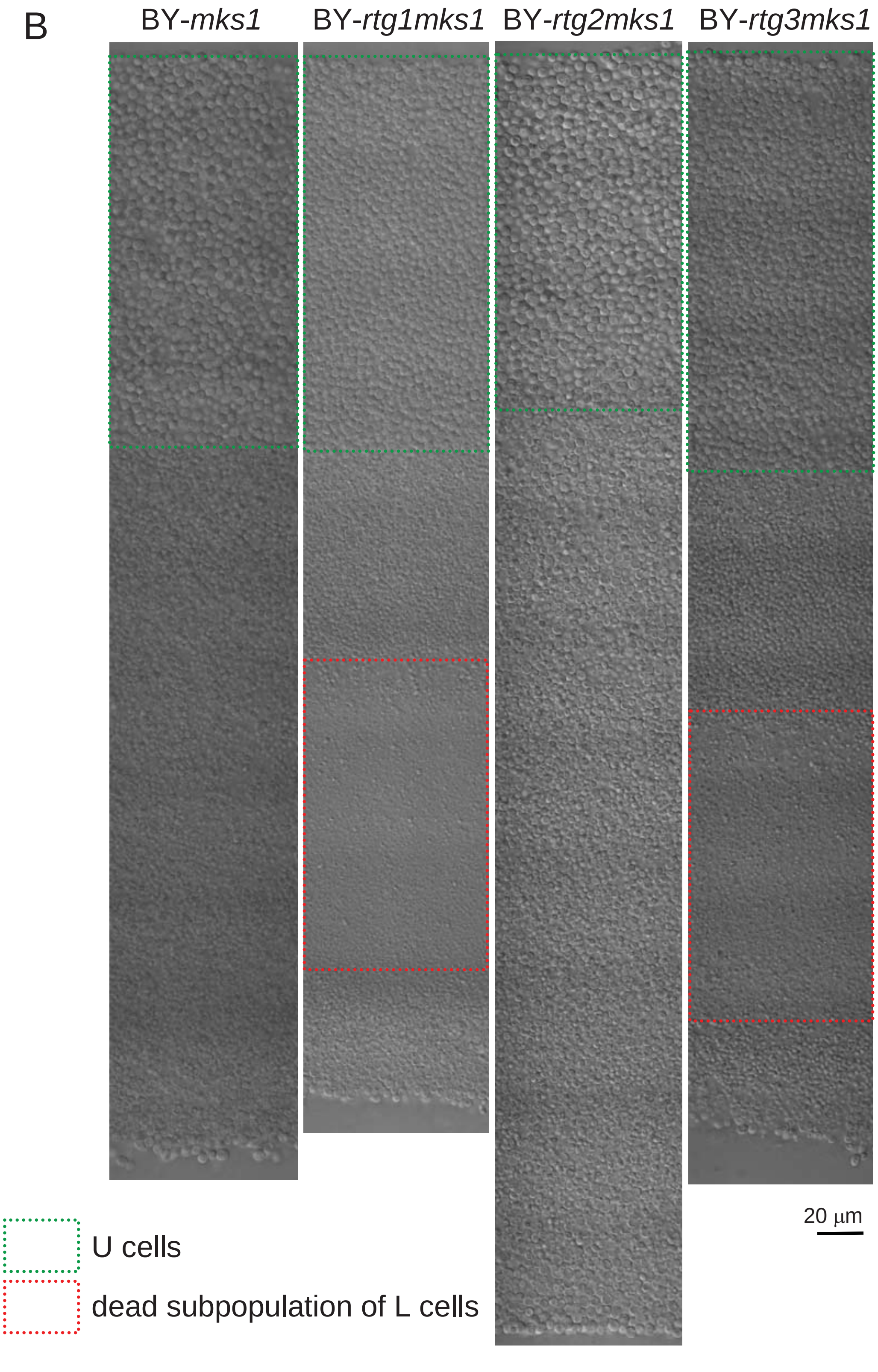

Figure S1

Vertical transversal cross-sections of 17-day-old colonies formed by wt and KO strains. (A, B) Cross-sections (20 µm thin) of colonies formed by wt and different *rtg*Δ strains were prepared by vibrating microtome and visualized by Nomarski contrast. The green dotted rectangle delimitates U cells, and the red dotted rectangle delimitates lower L cells with decreased viability in colonies of *rtg*Δ strains.
