# Supplementary material for: Divergent branches of mitochondrial signaling regulate specific genes and the viability of specialized cell types of differentiated yeast colonies
Source: Oncotarget. 2016 Mar 15;7(13):15299–314. doi: 10.18632/oncotarget.8084 (PMC4941242; doi:10.18632/oncotarget.8084)
Supplement: Supplementary file 2 [file oncotarget-07-15299-s002.pdf]

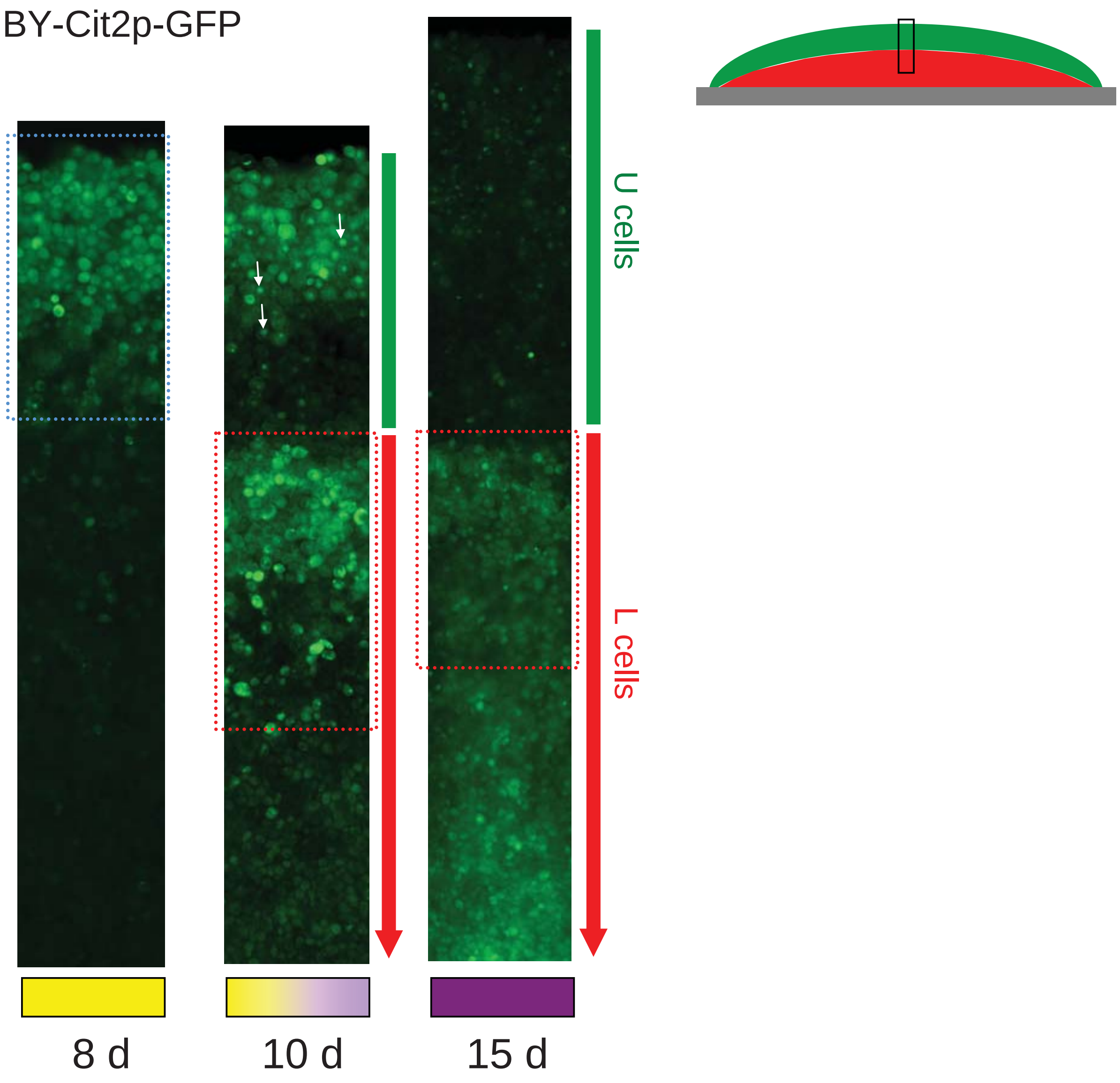

Figure S2

Vertical transversal cross-sections of giant colonies occurring in different developmental phases formed by BY-Cit2p-GFP strain. Cross-sections (20 μm thin) of colonies were prepared by vibrating microtome, and Cit2p-GFP fluorescence was detected by wide-field fluorescence microscopy. Sections of colonies occurring in late acidic phase (8-day-old, yellow bar), in the phase of transition from acidic-to-alkali phase (10-day-old, yellow-violet bar) and in the fully developed alkali phase (15-day-old, violet bar) are shown. The green bar indicates U cells, and the red bar indicates upper part of L cells. White arrows mark examples of cells with vacuolar GFP fluorescence, indicating Cit2p-GFP degradation by autophagy. The dotted blue rectangle delimitates upper cells of acidic phase colonies producing Cit2p-GFP. The dotted red rectangle delimitates upper L cells producing Cit2p-GFP. Fluorescence signal in lower parts of L cells is not related to Cit2p-GFP (as confirmed by WB, Figure 3) but to cellular autofluorescence with different spectral characteristics. Approximate position of the shown segment of cross-sections is depicted using a schematic view of a colony cross-section.
