# Supplementary material for: Divergent branches of mitochondrial signaling regulate specific genes and the viability of specialized cell types of differentiated yeast colonies
Source: Oncotarget. 2016 Mar 15;7(13):15299–314. doi: 10.18632/oncotarget.8084 (PMC4941242; doi:10.18632/oncotarget.8084)
Supplement: Supplementary file 3 [file oncotarget-07-15299-s003.pdf]

A

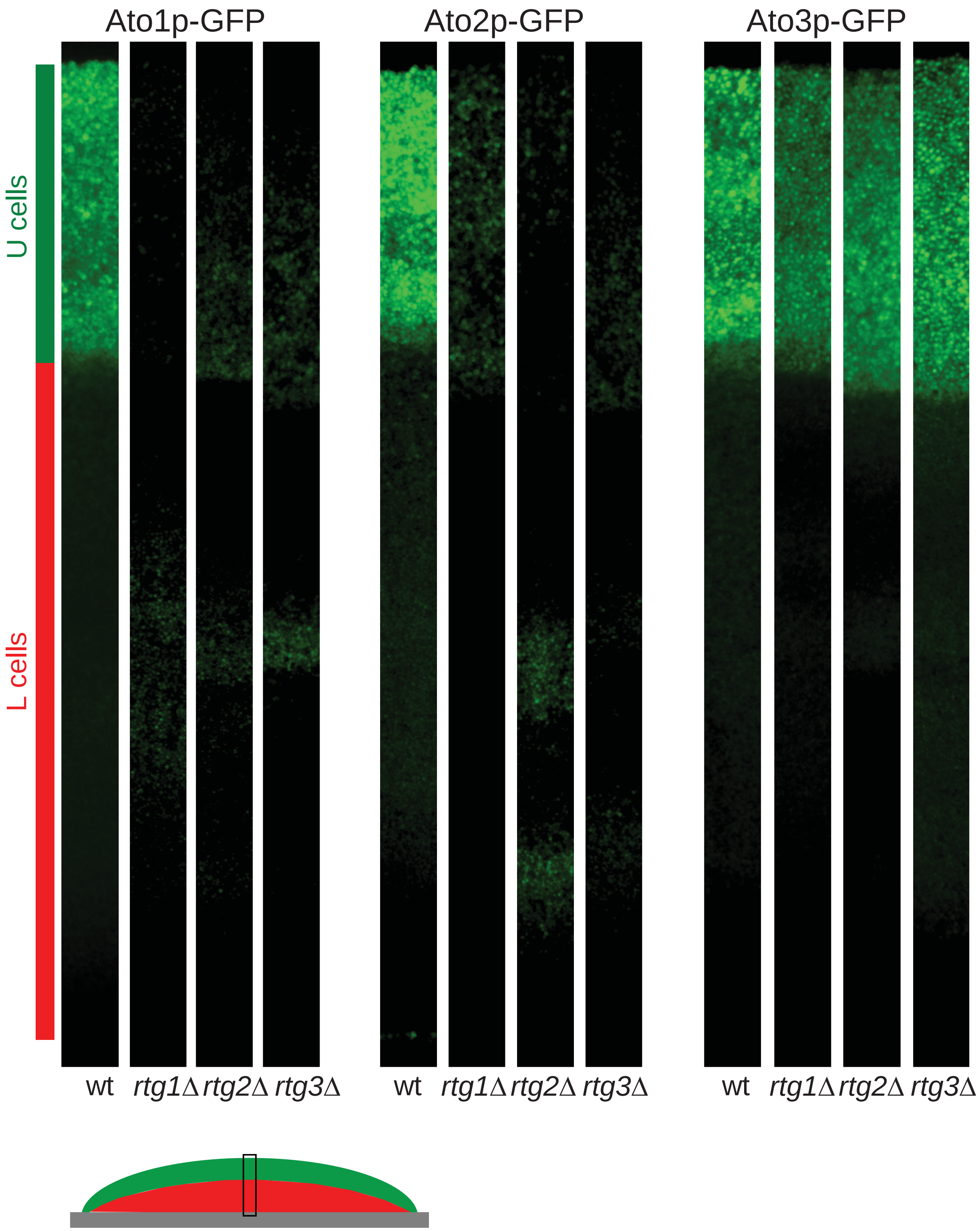

Ato1p-GFP
Ato2p-GFP
Ato3p-GFP
U cells
L cells
wt
rtg1Δ
rtg2Δ
rtg3Δ
wt
rtg1Δ
rtg2Δ
rtg3Δ
wt
rtg1Δ
rtg2Δ
rtg3Δ

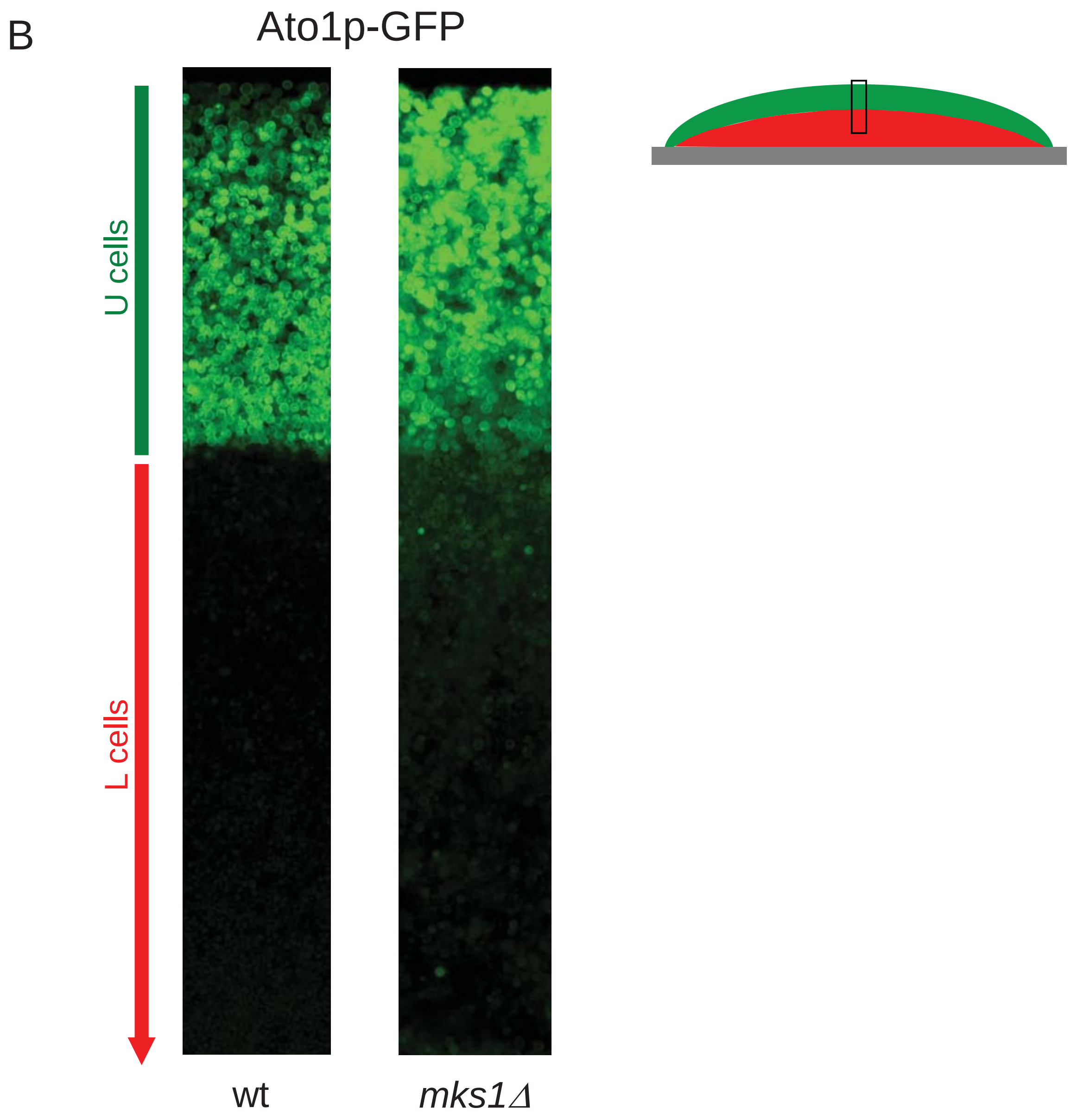

Figure S3

Vertical transversal cross-sections of 14-day-old colonies formed by wt and KO strains producing Ato-GFP proteins. Cross-sections (20 μm thin) of colonies formed by wt, *rtg*Δ and *mks1*Δ strains were prepared by vibrating microtome, and Ato-GFP fluorescence was detected by wide-field fluorescence microscopy. (A) Differences in Ato-GFP production in wt and *rtg*Δ colonies. Green and red bars indicate U and L cells, respectively. Approximate position of the shown segment of cross-sections is depicted below using a schematic view of a colony cross-section. (B) Differences in Ato1p-GFP production in wt and *mks1*Δ strains: U cells and upper part of L cells, showing that upper L cells in colonies of *mks1*Δ strain produce and properly localize Ato1p-GFP to the membrane. The same microscopy setup was used for wt and *mks1*Δ colonies. Approximate position of the shown segment of cross-sections is depicted on the right side using a schematic view of a colony cross-section.
