# Supplementary material for: Divergent branches of mitochondrial signaling regulate specific genes and the viability of specialized cell types of differentiated yeast colonies
Source: Oncotarget. 2016 Mar 15;7(13):15299–314. doi: 10.18632/oncotarget.8084 (PMC4941242; doi:10.18632/oncotarget.8084)
Supplement: Supplementary file 4 [file oncotarget-07-15299-s004.pdf]

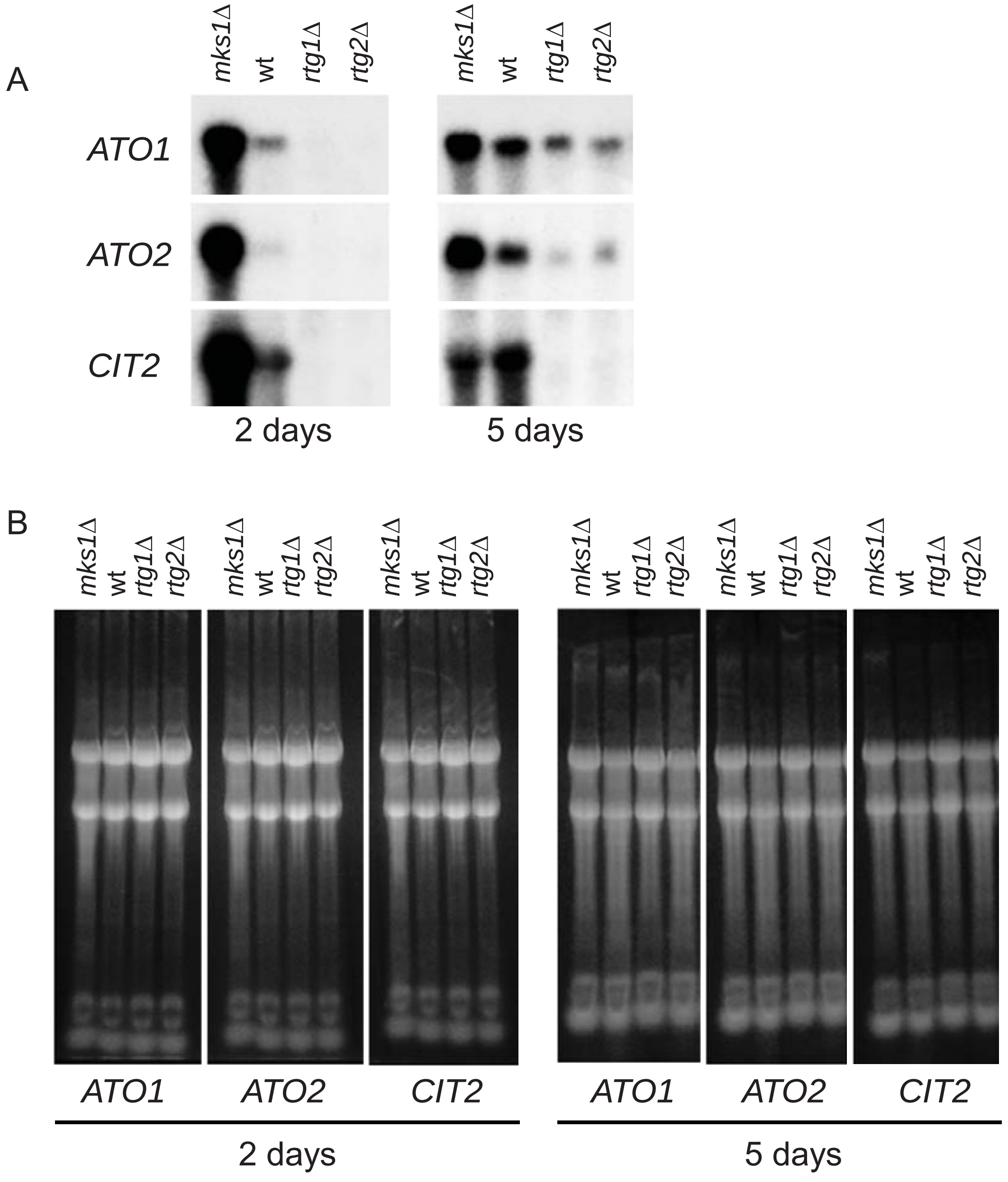

Figure S4:
***ATO1*, *ATO2* and *CIT2* mRNA levels in 2- and 5-day-old microcolonies of wt and KO strains.** A, Northern blots. B, rRNAs visualized by ethidium bromid staining (loading controls).
