# Supplementary material for: Divergent branches of mitochondrial signaling regulate specific genes and the viability of specialized cell types of differentiated yeast colonies
Source: Oncotarget. 2016 Mar 15;7(13):15299–314. doi: 10.18632/oncotarget.8084 (PMC4941242; doi:10.18632/oncotarget.8084)
Supplement: Supplementary file 5 [file oncotarget-07-15299-s005.pdf]

Loading controls for the Figure 3A

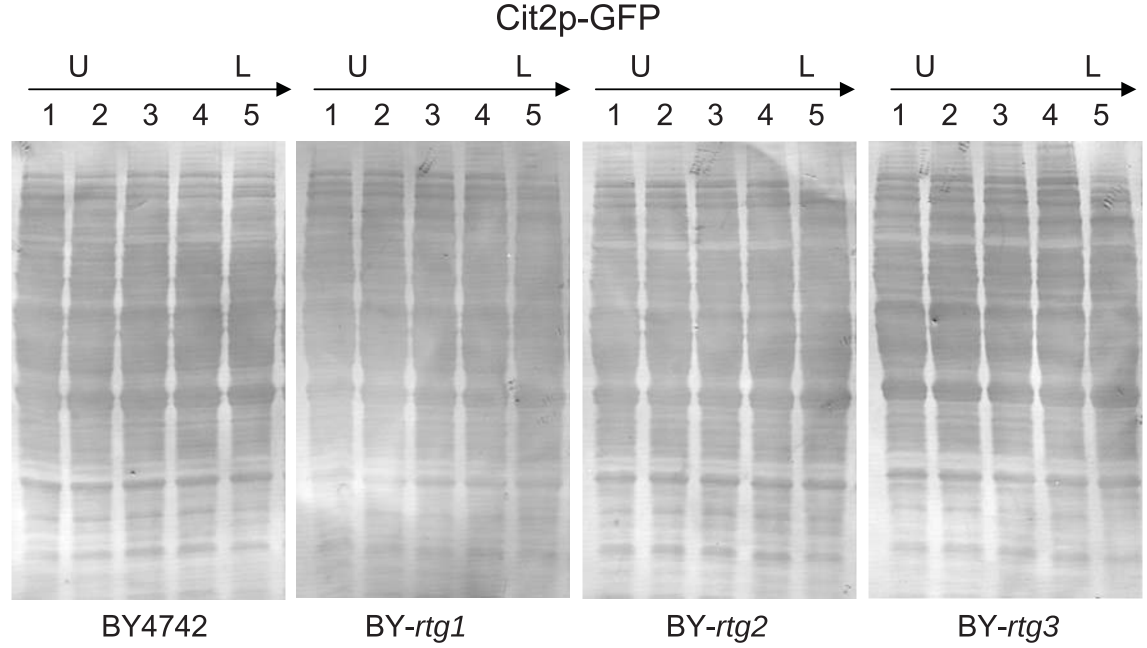

Loading controls for the Figure 3B

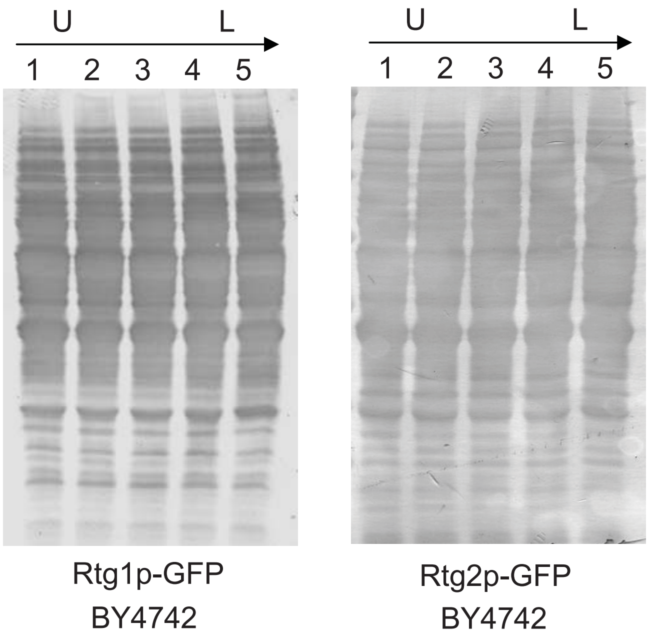

Loading controls for the Figure 4

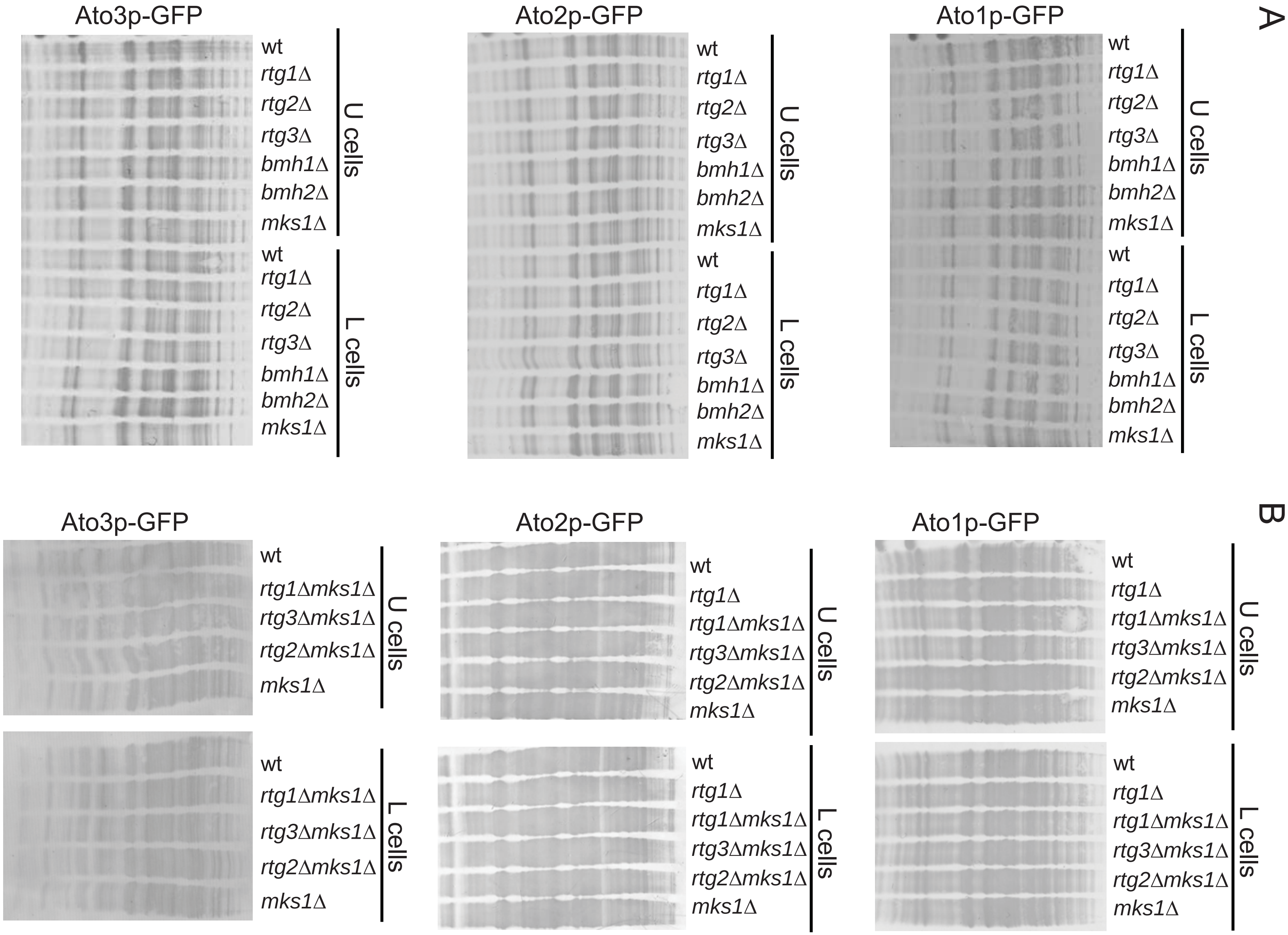

Loading controls for the Figure 5A

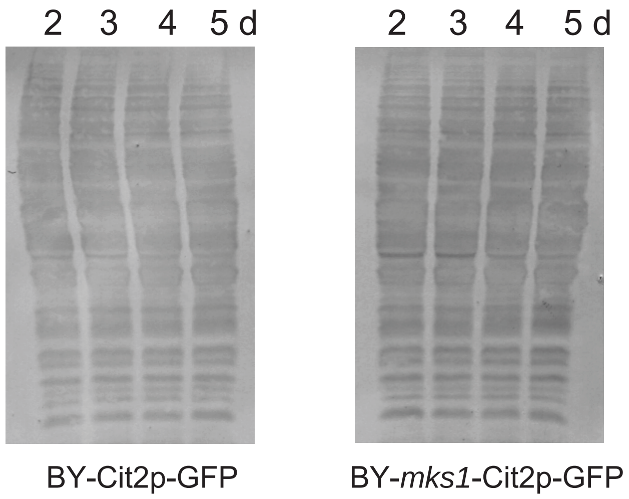

Loading controls for the Figure 5C

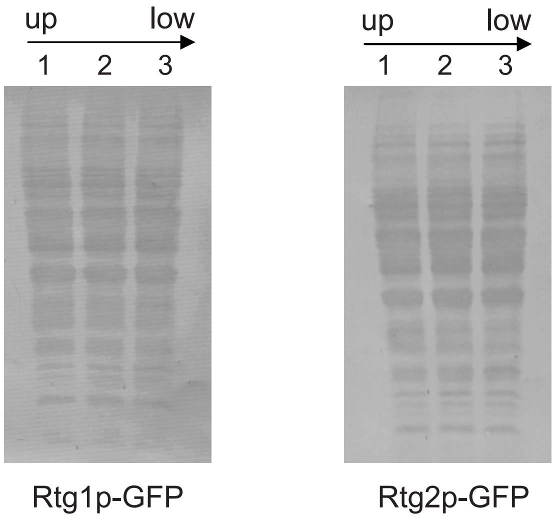

Figure S5
Loading controls for Western blots shown in Figures 3, 4 and 5.
Membranes stained by the Commassie blue dye.
